# Gastric Lavage in Acute Organophosphorus Pesticide poisoning (GLAOP) – a randomised controlled trial of multiple vs. single gastric lavage in unselected acute organophosphorus pesticide poisoning

**DOI:** 10.1186/1471-227X-6-10

**Published:** 2006-10-19

**Authors:** Yi Li, XueZhong Yu, Zhong Wang, HouLi Wang, XiangHuai Zhao, YuPing Cao, WeiZhan Wang, Michael Eddleston

**Affiliations:** 1 Department of Emergency Medicine, Peking Union Medical College Hospital, Chinese Medical Academy, Beijing, China; 2 Wen Shang Hospital, WenShang, Shan Dong Province, China; 3 First Central Hospital, BaoDing, He Bei Province, China; 4 Harrison International Peace Hospital, Hengshui, HeBei Province, China; 5 South Asian Clinical Toxicology Research Collaboration, Department of Clinical Medicine, University of Colombo, Colombo, Sri Lanka; 6 Scottish Poisons Information Bureau, Royal Infirmary, Edinburgh, UK

## Abstract

**Background:**

Organophosphorus (OP) pesticide poisoning is the most common form of pesticide poisoning in many Asian countries. Guidelines in western countries for management of poisoning indicate that gastric lavage should be performed only if two criteria are met: within one hour of poison ingestion and substantial ingested amount. But the evidence on which these guidelines are based is from medicine overdoses in developed countries and may be irrelevant to OP poisoning in Asia. Chinese clinical experience suggests that OP remains in the stomach for several hours or even days after ingestion. Thus, there may be reasons for doing single or multiple gastric lavages for OP poisoning. There have been no randomised controlled trials (RCTs) to assess this practice of multiple lavages. Since it is currently standard therapy in China, we cannot perform a RCT of no lavage vs. a single lavage vs. multiple lavages. We will compare a single gastric lavage with three gastric lavages as the first stage to assess the role of gastric lavage in OP poisoning.

**Methods/Design:**

We have designed an RCT assessing the effectiveness of multiple gastric lavages in adult OP self-poisoning patients admitted to three Chinese hospitals within 12 hrs of ingestion. Patients will be randomised to standard treatment plus either a single gastric lavage on admission or three gastric lavages at four hour intervals. The primary outcome is in-hospital mortality. Analysis will be on an intention-to-treat basis. On the basis of the historical incidence of OP at the study sites, we expect to enroll 908 patients over three years. This projected sample size provides sufficient power to evaluate the death rate; and a variety of other exposure and outcome variables, including particular OPs and ingestion time. Changes of OP level will be analyzed in order to provide some toxic kinetic data.

**Discussion:**

the GLAOP study is a novel, prospective cohort study that will explore to the toxic kinetics of OP and effects of gastric lavage on it. Given the poor information about the impact of gastric lavage on clinical outcomes for OP patients, this study can provide important information to inform clinical practice.

## Background

Deliberate self-poisoning has reached epidemic proportions in parts of the developing world where highly toxic poisons and sparse medical facilities ensure a high fatality rate. Pesticides are the major problem – the WHO estimates that they cause more than 250,000 deaths worldwide each year [1]. Most deaths are due to organophosphorus (OP) insecticides [2].

Gastric lavage is a routine first aid procedure for self-poisoned patients in the People's Republic of China and many Asia countries [3-6] but not in developed countries. Guidelines used in the West suggest that gastric lavage should only be used when two criteria are met: 1) the patient presents within an hour of poison ingestion and 2) the amount of toxin is substantial [7]. If patients present after one hour, the stomach contents are thought to have likely passed into the small bowel so that gastric lavage would not then be effective. In addition, if done carelessly, gastric lavage may push gastric contents beyond the pylorus, thereby enhancing absorption [7].

There is no evidence for the clinical effectiveness of gastric lavage. During 1997, the American Academy of Clinical Toxicology and European Association of Poisons Centres and Clinical Toxicologists published reviews assessing the value of a single gastric lavage on admission in acute self-poisoning [8]. The position statement noted that serious complications are associated with lavage and that there were no studies of sufficient quality with which to assess the clinical benefit of gastric lavage. The reports stressed the importance of establishing high quality RCTs with clinically relevant outcomes in order to determine the role of gastric lavage in poisoning management.

However, we should note that the evidence on which the European and American guidelines are based largely involves overdoses of relatively safe pharmaceutical agents in Western countries. It is not clear how relevant such studies are to rural Asia where OP pesticides are often ingested and the case fatality some 20–50 times higher than the West. Furthermore, it is easier to aspirate liquid pesticides than tablets because tablets can block the holes at the end of the lavage tube [9].

To determine if there are any papers relevant to liquid OP pesticide poisoning, we also carried out a Clinical Evidence search and appraisal during 2005, plus a systematic review of Medline, Embase, and Cochrane Collaboration databases, to identify relevant RCTs looking at gastric lavage in OP poisonings. We were unable to find any studies published in English or other European languages. We subsequently searched the Chinese National Knowledge Infrastructure. Although this identified over 500 papers describing lavage in OP poisoned patients, none reported RCTs. Overall, there is a lack of high-quality evidence on the effectiveness of single or multiple gastric lavages in acute human OP poisoning.

Current clinical practice in China is to give a single gastric lavage to all patients with OP self-poisoning according to the Chinese textbook [6]. Furthermore, the practice of giving multiple gastric lavages has become recently popular from several non-randomised controlled trials performed in China, despite the biases inherent in the study design. For example, You and colleagues showed that repeated lavages, every 2 hrs for 24–48 hrs, reduced mortality from 20.94% to 4.65% in a study of 86 patients [4]. Luo and colleagues looked at lavages every 4–6 hrs for up to 24 hrs and demonstrated a reduction in mortality from 47.5% to 14.6% [5]. Such studies, despite their methodological weaknesses, suggest that the practice of giving multiple gastric lavages must be assessed in a RCT.

The multiple lavage technique may work by removing pesticide left in the stomach after the first lavage, pesticide re-entering the stomach from the small bowel in the supine patient, and/or pesticide secreted by the gastric mucosa into the stomach (enterovascular circulation). Chinese studies have found the concentration of OP in stomach to be still high several hours or even days after ingestion and gastric lavage [10,11]. This contrasts with unpublished studies from Hanoi, Vietnam, which have found little OP to be present after gastric lavage (Dawson, personal communication).

Since gastric lavage is currently standard therapy in China, we are not able to perform a RCT of no lavage vs. a single lavage vs. multiple lavages. Instead, as a first step in a reassessment of the role of gastric lavage in OP poisoned patients, we have designed a trial comparing patients receiving standard therapy plus either one lavage or three lavages. If three lavages do not offer benefit over a single lavage, it may then be appropriate to consider designing a placebo controlled RCT of gastric lavage.

If three gastric lavages are shown to be beneficial, then such lavages should be encouraged worldwide for OP pesticide poisoning since the technique is cheap, widely available, and reasonably safe once the airway is protected.

## Methods/Design

The study is designed as an open RCT with two parallel groups: a single gastric lavage on admission vs three gastric lavages at four hour intervals from admission. Chinese adult patients presenting to a secondary hospital with a history of acute OP self-poisoning will be recruited to the study.

The trial has been drawn up and was designed to be compliant with the Consolidated Standards of Reporting Trials (CONSORT) statement.

### Patients

The RCT will be performed in the emergency department and/or the intensive care units (ICU) of the Harrison International Peace Hospital in Hengshui He Bei Province, the first Central Hospital in Baoding He Bei Province, and the Wen Shang Hospital in Shan Dong Province.

All patients with a history of OP pesticide ingestion will be approached concerning the study. Written informed consent will be requested from conscious patients by a study physician. Consent for unconscious adult patients will be sought from accompanying relatives. Patients who are unconscious on admission and present without relatives will not be recruited to the trial.

Patients who do not give consent to recruitment will receive usual care from the medical ward staff.

### Inclusion and exclusion criteria

The study aims to recruit all patients admitted to the adult medical wards of the study hospitals with a history of acute oral OP pesticide self-poisoning 12 hours within ingestion requiring atropine. Exclusion criteria will be: age under 18 yrs; known pregnancy; co-ingestion of another poison; and previous recruitment to the RCT.

### Patient management

**Figure 1:**
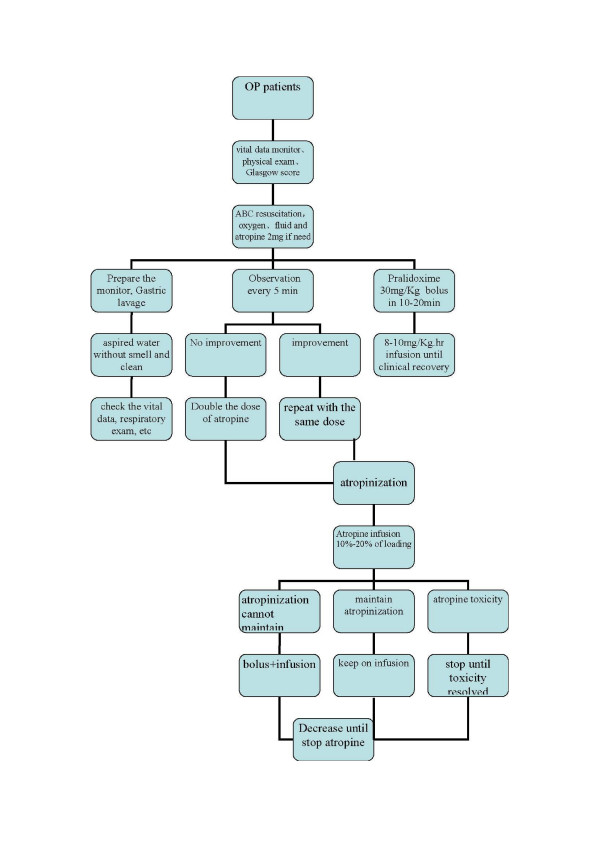
Treatment protocol for OP poisoned patients.

All patients will be seen on admission to the emergency department and/or ICU wards, and resuscitated as necessary by ward doctors together with study doctors. Standard treatment will be administered as described in figure 1[12].

We will use five markers for the routine assessment of atropine requirements: miosis, sweating, poor air entry into the lungs due to bronchorrhoea and bronchospasm, bradycardia, and hypotension. If one of the markers is present, we will give a 2 mg bolus of atropine. Markers of sufficient atropine administration are a clear chest on auscultation with no wheeze, heart rate >80 beats/min, pupils no longer pinpoint, dry axillae and systolic blood pressure >80 mmHg. If there is no improvement in five minutes, the dose will be doubled and then doubled again each time there is no response. The aim of atropine therapy is to clear the chest and reach the end-points for the chest, heart rate, and blood pressure. Once the patient is atropinized, 10–20% of the total amount of atropine required to load the patient will infused per hour, titrated to the patient's condition.

Pralidoxime chloride will be given according to the protocol recommended by the World Health Organization, i.e. a loading dose of 30 mg/kg over 20 min, followed by a continuous infusion of at least 8–10 mg/kg per hour until clinical recovery (for example 12–24 hours after atropine is no longer required or the patient is extubated) or 7 days.

Lavage will be done as soon as a patient is stabilised and resuscitated as above. Patients will receive gastric lavage on admission, regardless of whether they have already received it at another health centre.

Patients will remain under the care of the hospitals' consultant physicians using management protocols agreed between the consultants and study team. The ward medical teams will make decisions about intubation and transfer of patients to intensive care or for cardiac pacing independently of study doctors. All decisions are made on the basis of clinical condition and do not reflect the poison ingested or the times of lavages.

Patients will be seen regularly by doctors at least every three hours and more often according to clinical need 24 hrs a day. Patients will also seen on a study ward round twice each day (0830, 2030) at which time their condition is recorded. Significant events (intubation, seizures, death) will be recorded at the time of the event. The patients' condition over the previous 12 hrs will be reviewed at each ward round.

Patients will be first managed on the medical ward. Seriously ill patients, as judged by the medical team, will be transferred to ICU as a bed becomes available.

Criteria for intubation include respiratory rate of less than 10 breaths/minute, abdominal breathing, failure of non-invasive methods to maintain a patent airway, or the fraction of oxygen pressure to its concentration is lower than 300. Hypotensive patients, unresponsive to atropine and fluid resuscitation, are treated with dopamine plus dobutamine as necessary.

### Trial interventions and study procedures

Patients will be placed in left lateral position and a nose-gastric tube placed through a nostril. Once in the stomach, its position will be confirmed by aspirating gastric content and auscultating over the stomach. The gastric content will be sucked out first and then 300 ml of water at room temperature pushed in. The water will then be aspirated completely and another 300 ml administered. The above procedures will be repeated until the aspirated water is without smell and clear – this end point will be judged by at least two doctors.

The above procedure will be repeated 4 hours and 8 hours after the first gastric lavage in the group of patients receiving multiple lavages. 24 hours after admission, and after gastric lavage and sample collection has been finished in all groups, the gastric tube will be pulled out.

The procedure will be done by skilled medical staff. Vital body signs, including ECG (electric cardiac graph) monitor, blood pressure, heart rate, respiratory rate, and pulse oxygen saturation will be monitored throughout the lavage. Patients will also be monitored for complications such as aspiration. There should be no major problem with compliance since the lavage will be given by the study team.

A 10 ml blood sample will be taken using a sterile syringe and needle from each patient on recruitment. Further 5 ml blood samples will taken at one, four and twelve hours post-treatment, and then at daily intervals until discharge or death, from a subset of patients. Whether a needle or indwelling cannula is used will be determined by the wishes of the patient. The identity and blood concentration of the poison will be assayed retrospectively in a subset of patients due to limited laboratory facilities.

A 10 ml sample of gastric aspirate will be taken at the beginning of each gastric lavage so that the concentration of pesticide present in the stomach can be calculated. The volume of fluid aspirated before the first 300 ml of water is given will be recorded.

### Allocation of patients

Patients will be randomised to one of two study arms. The random allocation sequence will be generated by computer and incorporated into a Microsoft Access programme written for patient recruitment, randomisation and event recording. Stratified block randomisation will be performed according to patient status on admission (Glasgow coma score 14–15/15, Glasgow coma score <14/15).

The allocation sequence will be generated independently by the study statistician and programmer, who will have no role in patient recruitment, treatment or assessment. Variable block sizes of 3, 6 and 9 will be used to allocate patients in equal numbers to each treatment group i.e. ratio 1:1.

Participants will be recruited and randomised by a study doctor at the bedside using a dedicated handheld computer at each study hospital. Randomisation will occur after the patient's baseline data have been entered and receipt of consent noted, and cannot be manipulated by study doctors. The recruiting doctor will be unable to predict allocation before randomisation.

All practical steps have been taken to avoid bias: (i) the randomisation program has been designed to be rapid and simple to operate, and yet remain independent of the investigators; (ii) the next treatment allocation cannot be predicted in advance; (iii) the primary outcome, vital status at discharge, is unambiguous, and the secondary outcomes are objective; (iv) all outcomes are recorded by the study team, not other hospital physicians;

(v) patient follow-up should be near 100% complete; and (vi) the analysis will be performed on an intention-to-treat basis.

### Outcomes

The primary outcome is all-cause mortality during hospital admission.

Secondary outcomes include proportion of patients requiring intubation for respiratory failure; period of ventilation; proportion of patients developing the intermediate syndrome; and 50% recovery time for red cell acetylcholinesterase enzyme activity.

### Sample size

In all three patient enrolled hospitals, at least 15% of poisoned patients die before discharge[6]. An absolute reduction of 6% will be clinically important. In order to be able to detect whether multiple gastric lavage reduces the death rate from 15% to 9%, with a significance level (alpha) of 5% and a power of 80%, a minimum of 454 patients must be recruited to each arm of the trial (i.e. 908 patients in total).

There should be no loss to follow up for the primary outcome. Patients will either be discharged alive from the wards or their bodies transferred to the morgue for judicial autopsy. Secondary clinical outcomes will be assessed in hospital before patient discharge; red cell AChE will be measured in a subset of patients by a laboratory blind to allocation.

### Study hypotheses and planned analyses

The main hypothesis is that multiples gastric lavages will reduce the case fatality rate from 15% to 9%, hence the primary comparison will be of multiple lavages with a single lavage.

It is possible that multiple lavages will be more effective the earlier they are started. Therefore we will assess the trends in clinical effectiveness according to time post-ingestion to start of therapy. In order to determine whether treatment should be started irrespective of severity, we will also assess trends in case fatality rates across a gradient of severity.

Subgroup analyses are planned to look at the consistency of treatment effect across particular OPs that are common in China, eg. dichlorvos, phorate, dimethoate. Analyses will also be done to check whether ingestion of a meal within one hour of the ingestion affects outcome.

Admission blood and stomach content samples will be retrospectively analysed to determine the identity of the poison ingested. The primary analyses will then be repeated with correction for the identity of the poison. The time-dependent variations of OP level will be analysed in order to provide OP toxic kinetic data.

### Statistical analysis

The main analysis will be carried out on an intention-to-treat basis, using the chi squared test for the primary outcome (or Fisher's exact test if appropriate) and for other dichotomous outcomes. Relative risk (plus risk reduction), absolute risk reduction and number needed to treat (plus 95% confidence intervals) will also be calculated. For outcomes where time-to-event is recorded, the log rank test will be used to compare treatment groups. In addition, a Kaplan-Meier curve will be produced to illustrate the comparison of mortality between groups.

An analysis of trends in treatment effect for factors 'reported time from ingestion to treatment' and 'patient status on admission' will also be performed using statistical modelling techniques.

### Data Monitoring and Ethics Committee (DMEC)

An independent DMEC has been established for the trial. For the duration of recruitment, interim analyses will be supplied by the trial statistician, in strict confidence, to the DMEC, together with any other analyses the DMEC may request. The data will be supplied to the Chair of the DMEC as often as he requests. Meetings will be arranged periodically, as considered appropriate by the Chair. In the light of interim data, and other evidence from relevant studies, the DMEC will inform the principal investigator (Dr Li Yi), if in their view (i) there is proof beyond reasonable doubt that the data indicate that any part of the protocol under investigation is clearly indicated or contra-indicated, either for all participants or for a particular subgroup of trial participants, or (ii) it is evident that no clear outcome will be obtained.

The decision to inform the principal investigator in either of these circumstances will, in part, be based on statistical considerations. Appropriate criteria for proof beyond reasonable doubt cannot be specified precisely. A difference of at least three standard deviations in the interim analysis of the major endpoint may be needed to justify halting, or modifying, such a study prematurely. If this criterion were to be adopted, it would have the practical advantage that the exact number of interim analyses would be of little importance, and so no fixed schedule is proposed [13]. Unless modification or cessation of the protocol is recommended by the DMEC, the principal investigator, co-investigators, collaborators and administrative staff will remain ignorant of the results of the interim analyses. Collaborators and all others associated with the study may write to the Chair of the DMEC to draw attention to any concern they may have about the possibility of harm arising from the treatment under study, or any other matter that may be relevant. The principle investigator will follow the advice of the Chairman concerning decisions to continue or stop the trial.

The members of the DMEC for this study are:

Dr Bin Du, Dept of Medicine Intensive Care Unit, Peking Union Medical College Hospital (Chairman)

Professor Andrew Dawson, Dept of Medicine, University of Peradenya, Sri Lanka

Professor Abul Faiz Dept of Medicine, Dhaka Medical College, Bangladesh

Professor Lau Fei Lung, Hong Kong Poison Control Center, Hong Kong, China

Dr. ShaoMei Han, Dept of Statistics, Peking Union Medical College Hospital (Statistician)

Professor Ji Jiang, Dept Pharmaceulogy, Peking Union Medical College Hospital

## Discussion

The GLAOP study is a multi-site, prospective cohort study that seeks to evaluate the impact of gastric lavage, particular OP and ingestion time etc. on a variety of important outcomes. Whereas previous studies of gastric lavage have measured the outcomes in drug overdosers[8], the GLAOP study is distinguished by its large sample size of OP patients and its comprehensive measurement of poison levels. Building on these strengths allows the GLAOP study to evaluate the impact, and associated mechanisms of gastric lavage action on longer-term patient outcomes, and to generate hypotheses for future research. Ultimately, the GLAOP study seeks to inform clinicians about the gastric lavage therapy in pesticide poisoning, so as to facilitate change in clinical practice and improve outcomes for pesticide poisoning patients. The GLAOP study has potential limitations. First, besides times of gastric lavage, there are many known factors influencing the result. These include ingestion time, particular OP, amount of ingestion, the underlying disease and so on. Furthermore some important factors may be unknown or unmeasured, resulting in residual confounding and bias. If these differences are associated with the outcomes of interest, the study results may be difficult to analyze. Fortunately, software for the randomization has been used in the triage of the trial. Second, because of the ethic concern, group without lavage at all can not be set up in China to investigate its role in OP patients directly. No matter what result was put out, single lavage better or multiple lavages better, it is difficult to put forward the trial further because of the different ethic concerns in the world. For example, if multiple lavage shows no better than single lavage, it is a long journey to set up a RCT to explore the difference between single lavage and no lavage in China. Third, although drawn from 3 different medical centers and a treatment protocol has been used, all these hospitals are in China; thus, the GLAOP study results may not generalize to OP patients in other settings.

These limitations suggest several directions for future studies. First, the design of future randomized trials of gastric lavage. Narrow the inclusion criteria of OP patients, then the respective factors can be studied better. Second, in order to put forward relative studies thereafter, communications with experts in the world are necessary. Winning the understanding of the trial and its importance, it will be better to accomplish the trials. Finally, additional observational studies especially taken outside of China should be conducted to assess the generalizability of findings from the GLAOP study.

In summary, the GLAOP study is a prospective cohort study that seeks to provide new knowledge about the association of gastric lavage, particular OP and other aspects of OP with the patient outcomes. Strengths of the study include comprehensive measurement of samples and outcomes, and a relatively large projected sample size. Results from the GLAOP study should help to explore to the toxic kinetics of OP and effects of gastric lavage on it, help to improve the care and outcomes of OP patients.

## Abbreviations

GLAOP = gastric lavage in acute organophosphorus pesticide poisoning; OP = organophosphorus; RCT = randomised controlled trials; ICU = intensive care unit; ECG = electric cardiac graph; DMEC = data monitoring and ethics committee.

## Competing interests

The author(s) declare that they have no competing interests.

## Authors' contributions

YL, ME, and XY were responsible for identifying the research question, and contributing to drafting of the study protocol. ZW, HW, WW, YC and XZ have all contributed to the development of the protocol and study design. YL and ME were responsible for drafting of this paper; all authors have provided comments on drafts and have read and approved the final version.

## Pre-publication history

The pre-publication history for this paper can be accessed here:



## References

[B1] The impact of pesticides on health: preventing intentional and unintentional deaths from pesticide poisoning. http://www.who.int.

[B2] Eddleston M (2000). Patterns and problems of deliberate self-poisoning in the developing world. QJM.

[B3] Gu YL, Wan WG, Xu ML, Zhou HJ (2004). Gastric lavage for OP pesticides poisoning patients. Zhonghua Lao Dong Wei Sheng Zhi Ye Bing Za Zhi.

[B4] You RH, Zheng Q (2002). The experience of repeated gastric lavage in acute severe OP pesticides. SiChuan Medicine.

[B5] Luo QH, Liao LQ, He B, Liao J, Lu WH (2002). The observation of repeated gastric lavage in acute severe OP pesticides. SiChuan Medicine.

[B6] Chen HZ Pesticide Poisoning. Practice of Internal Medicine. People's Medical Publishing House.

[B7] Jones AL (2000). Letters to managing self poisoning. BMJ.

[B8] Vale JA (1997). Position statement: gastric lavage. American Academy of Clinical Toxicology; European Association of Poisons Centres and Clinical Toxicologists. J Toxicol Clin Toxicol.

[B9] Grierson R, Green R, Sitar DS, Tenenbein M (2000). Gastric lavage for liquid poisons. Ann Emerg Med.

[B10] Zhang DY, Liu YF, Fu FH, Lu JQ, Chen CE, Jia SH (1998). Time phase of the relics of OP in stomach after one time lavage. J BinZhou Med College.

[B11] Liu YF, Fu FH, Zhang DY, Li XH, Han JT (1997). The relationship between the concentration of blood and gastric contents and the activity of acetylcholinesterase enzyme in OP patients. Zhonghua Nei Ke Za Zhi.

[B12] Eddleston M, Dawson A, Karalliedde L, Dissanayake W, Hittarage A, Azher S, Buckley NA (2004). Early management after self-poisoning with an organophosphorus or carbamate pesticide – a treatment protocol for junior doctors. Crit Care.

[B13] Peto R, Pike MC, Armitage P, Breslow NE, Cox DR, Howard SV, Mantel N, McPherson K, Peto J, Smith PG (1976). Design and analysis of randomised clinical trials requiring prolonged observation of each patient. I. Introduction and design. Br J Cancer.

